# Unmet family planning needs among female refugees and asylum seekers in Germany – is free access to family planning services enough? Results of a cross-sectional study

**DOI:** 10.1186/s12978-020-00962-3

**Published:** 2020-07-29

**Authors:** Melisa Guelhan Inci, Nadja Kutschke, Sara Nasser, Sara Alavi, Ingar Abels, Christine Kurmeyer, Jalid Sehouli

**Affiliations:** 1 Department of Gynecology, Campus Virchow-Klinikum, Charité – Universitätsmedizin Berlin, corporate member of Freie Universität Berlin, Humboldt-Universität zu Berlin, and Berlin Institute of Health, Augustenburger Platz 1, 13353 Berlin, Germany; 2 Mentoring Competence Center, Campus Charité Mitte, Charité – Universitätsmedizin Berlin, corporate member of Freie Universität Berlin, Humboldt-Universität zu Berlin, and Berlin Institute of Health, 10117 Berlin, Germany; 3 Women and equal opportunities officer, Campus Virchow-Klinikum, Charité – Universitätsmedizin Berlin, corporate member of Freie Universität Berlin, Humboldt-Universität zu Berlin, and Berlin Institute of Health, 13353 Berlin, Germany

**Keywords:** Germany, Refugee health, Family planning, Women, Contraceptive use, Pregnancy, unplanned

## Abstract

**Background:**

After the 1968 United Nations International Conference on Human Rights, access to family planning services became a human right. Such a service is of central importance to women’s empowerment and is empirically needed to provide adequate healthcare. For registered refugees and asylum seekers in Germany complementary family planning services, including all forms of contraception, are free of charge. Yet, the success of these services remains unclear. The aim of this study is to describe the current reproductive health status of female refugees and to provide an initial overview of their existing unmet family planning and contraception needs.

**Methods:**

Over the course of 2 years, from December 2015 to December 2017, a set of 50 female-only discussion groups were conducted in community shelters for registered refugees in Berlin. A total of 410 women between the ages of 14 and 74 participated. A convenience sampling strategy was then applied and a total of 307 semi-structured questionnaires covering 41 items related to demographic data and women’s health were distributed to volunteering female participants over the age of 17. The statistical analysis of the questionnaires was performed using SPSS (IBM, PASW, Version 24). *P*-values less than or equal to 0.05 were considered statistically significant.

**Results:**

Of the 307 participants, the majority were from Syria and Afghanistan (30% respectively). The mean age was 33 years (range: 18–63). On average, each woman had 2.5 births (range: 0–10). Twenty-four women (8%) were pregnant and fifty-four of the women (18%) were trying to become pregnant. The majority of women were classified as “requiring contraception” (*n* = 195; 63%) of which 183 gave further information on if and how they used family planning methods. The calculated unmet need for family planning in this group was 47%. Of the remaining 53% of the women who used contraception, many utilised “traditional” methods (34% withdrawal method; 8% calendar method) which have a pearl index of 4–18 and can therefore be classified as rather insufficient birth control methods. Intrauterine contraceptive devices were used by 30%.

**Conclusion:**

Our study revealed that despite the provision of complementary family planning services, there remains unmet family planning and education needs in the female refugee community in Berlin. This study indicates that there is a major access gap to these services. Further research needs to be carried out to evaluate the access gap and clearly identify and implement action plans to address possible causes such as language barriers, lack of childcare and traumatic experiences.

## Plain English summary

The Unmet need within the provision of family planning services, defined as non-use of contraception by women of reproductive age who require it, has potentially severe implications at the individual, familial, community, and global level. Although there is a rising number of female refugees availing of family planning services free of charge in Germany, little research has been done to evaluate the success of this. Thus, this study seeks to record and evaluate the demand, and actual use, of contraception among fertile refugee women in Berlin in order to determine the extent of unmet family planning.

“Charité - Women for Women” conducted women-only discussion groups on female health in refugee shelters in Berlin and distributed questionnaires on gynecological and reproductive health to participants over the age of 17 years.

A total of 307 refugee women volunteered to take part in the study of which, the majority came from Syria and Afghanistan. Most of the participants were young and fertile and without the wish to become pregnant any time soon. Despite this, only 50% were using any kind of contraception. Once more, many of these women used non-reliable “traditional” methods. When a more reliable method was chosen, intrauterine devices were the most commonly used option.

On average, each woman already had 2.5 births. Eight percent were pregnant and 18 % were trying to become pregnant.

This survey underlines the enormous unmet need for family planning and education among female refugees. Furthermore, it highlights an existing access gap to services which are already in place.

## Introduction

Globally, many people have been forcibly displaced because of war, political oppression, violence and poverty. In 2016, a record high number of people were recorded to be refugees with the total number coming to 65.6 million [1]. In the same year, the number of asylum applications in Germany increased from 127,023 to 745,545 compared to 2013 with a third of the registered refugees being women between the ages of 11 and 65 [2]. In the following year, this ratio increased to 39.5% [3] and reached 43.3% in 2018 with a continuing upward trend [4]. With this increase in mind, Germany accounted for almost a third of all first-time applications (28%) registered in Europe [5].. Due to current geopolitical conflicts, a decline in the number of refugees is not expected in the near future, posing major challenges to the social and healthcare systems of the host countries.

In the literature, there is no extensive knowledge about the particular needs of this heterogeneous group of women, especially within the fields of healthcare and family planning [6]. Currently, the majority of research on registered refugees and asylum seekers in Germany is concerned with psychological questions and looks to the likes of Post-Traumatic Stress Disorders [7]. In terms of the reproductive health of refugees, a small body of research analyzes sexually transmitted diseases and general gynecological infections [8, 9] and describes the differences in antenatal and perinatal birth management [10]. Other studies offer an explanation to why there may be low health service utilization within migrant and refugee populations One such explanation can be accredited to the fact that many women flee with children and therefore pay less attention to their own health and well-being [7]. Furthermore, a lack of public awareness and health education is seen to be another contributing factor [11, 12]. Moreover, the perception of fertility, health and illness varies widely among refugees, which can be another major explanation for low engagement with healthcare and consulting services [13–15]. Additionally, access to interpreters and the general bureaucratic complexity of the German healthcare system is likely to hinder refugee women from receiving the care and health education they need [16].

Compared to those born and raised in Germany, refugees often have not had the privilege of formal sexual education that covers topics like contraception, pregnancy, and routine health screenings [17, 18]. Nonetheless, by German law registered asylum seekers are entitled to contraception. This applies to refugees who have already been granted refugee status as well as those who are still awaiting a decision [19]. Yet, the coverage of costs for contraception is state-specific. According to section 6 (§6) of the AsylbLG, “asylum seekers are reimbursed for contraceptives after prior application to the Social Welfare Office”. In Berlin specifically, asylum seekers with granted asylum status and those still awaiting a decision, can receive free counseling at sexual health and planning centers, where all forms of contraceptives are free of charge. However, there is currently no data on the use and the acceptance of any of these services among the refugee population.

The objective of this study is to record and evaluate the demand, as well as the actual use of contraception, among refugee and asylum-seeking women of childbearing age in Berlin and to determine the extent of unmet need for family planning. As per the WHO criteria, the term “unmet need for family planning” describes women who wish to stop or delay childbearing but are not using any method of contraception. In addition to this definition, data around other aspects of reproductive health such as birth-rate and conceptual behavior are collected in order to understand the extent of the unmet need for family planning. This data can have a significant impact at individual, family, community and global level [20]. Consequently, this study may provide much needed information for the development of legislation by politicians as well as providing a basis for clinical guidelines for physicians to improve patient centered care and integration programs [21].

## Methods

### Discussion groups and sampling strategy

From December 2015 to December 2017, “Charité’s Women for Women” conducted 50 educational evenings in the form of multiple 3-h long discussion groups in government-funded refugee housing centers across the city. The selection of refugee accommodation was based on a publicly accessible list of the “Landesamt für Flüchtlingsangelegenheiten” in Berlin. In the shelters, female gynecologists, with the assistance of medical language interpreters, gave presentations about women’s health to a total of 410 female asylum seekers and refugees between the age of 14 and 74. Each presentation included information on contraceptive options, breast cancer self-examination and maternal health within the German healthcare system. The events were for women only, in order to build trust within the group and provide privacy during the study questionnaire. Furthermore, in order to ensure that all women had the chance to participate, the management organized childcare for the children. Following the event, each woman was given the option to complete a survey. A convenience sampling strategy was applied and a total of 307 agreed to take part in the study. Semi-structured questionnaires covering both demographic data and questions about general and more specific women’s health issues were then distributed to the volunteering women over the age of 17.

### The survey

The questionnaire had 41 items covering age, country of origin, education status, previous births, further desire for children, and especially the use of contraception. Furthermore, chronic disease, gynecological infections, oncological history and physical and emotional trauma were part of the survey. Multiple answers were possible.

Qualified interpreters translated the complete questionnaire into multiple languages (Arabic, Farsi, Russian, English and Albanian). Prior to commencing the project in 2015, pilot survey questionnaires were evaluated to ensure no linguistic misunderstandings would arise.

The survey was distributed on paper and conducted individually. Trained interpreters provided assistance with completing the survey when requested. This was carried out in a private setting to maintain confidentiality among the participants. Each participant gave written consent prior to completing the survey. In cases of functional illiteracy, interpreters read all written material (questionnaire, study information, declaration of consent) aloud with the participant in a private setting.

Due to the sensitive nature of the topics and the vulnerability of our respondents, we refrained from including a direct question regarding their sexual activity in the questionnaire. Instead, women were regarded as sexually active when they stated at least one of the following: currently in an active relationship, pain during sexual intercourse, wish to become pregnant within the next 12 months or when they did not mention virginity.

### Group allocation

The target group was women over the age of 17 years old. During the evaluation the women were divided into two groups. Group 1: women with no need for family planning (menopausal, not sexually active, pregnant or women currently wanting to become pregnant) and group 2: women in need of family planning (sexually active, fertile women with no wish to become pregnant any time soon).

Group 2 was further divided into two subgroups: Subgroup 2a: women using contraception (contraceptive prevalence) and subgroup 2b: women not wanting to become pregnant but not using contraception (unmet family planning need). Both subgroups conform with the WHO definition of contraceptive prevalence and unmet need for family planning [22].

The subgroups were then compared according to age, education level, sexual education and time living in Germany. In order to evaluate the different contraception practices of the participants in subgroup 2a, the different contraception methods were categorized as either modern (birth control pills, IUDs, condoms and sterilization) or traditional (withdrawal method/coitus interruptus and calendar method). The Pearl Index was then used to determine the efficacy of different methods: Modern methods have a pearl index between 0.1 and 12 (birth control pill: 0.1–0.9; IUDs: 0.16–0.8; condom: 2–12); traditional methods between 4 and 18 (coitus interruptus: 4–18; sterilization: 0.1–0.3) [23].

### Statistical analysis

The statistical analysis of the questionnaires was performed using SPSS (IBM, PASW, Version 24). All clinical and demographic parameters were summarized in descriptive statistics. The chi-square test was used for testing categorical data between groups. Kruskal-Wallis one-way analysis of variance was used to compare continuous data. *P*-values less than or equal to 0.05 were considered statistically significant.

### Ethic board

The sensitive nature of the topic and the vulnerability of the respondents have been taken into great consideration. The institutional ethics review board at the Charité University Hospital Berlin approved the study.

## Results

### Demographics

Sociodemographic data on the study group can be seen in Table 1. The mean age of the 307 women taking part in the study was 33 years (range: 18–63). The majority of respondents were from Syria (29.6%) and Afghanistan (29.3%). The remaining women fled from Iraq (12.4%), Iran (11.1%), Albania (3.3%), Egypt (2%), Kosovo (1.3%), and other countries (5.2%: Yemen, Moldova, Chechnya, Serbia, Bosnia, Eritrea, Armenia, and Sudan). The remainder (5.9%) did not specify their country of origin. The participants reported a wide spectrum of educational backgrounds, with one third having either completed a vocational training program or a bachelor’s degree. Of the women, 141 (46%) reported having a diagnosis of at least one chronic disease, with 84 women (27%) reporting depression, 32 (10%) reporting hypertension, and 19 (6%) reporting type 2 diabetes.

**Table 1:** Demographic characteristics (*n* = 307)

Country of origin		Age in years	Years attended school	Completed bachelor’s degree	Vocational training
	n (%)	mean	mean	n	n
**Total**	**307 (100)**	**33**	**7,4**	**67 (22%)**	**30 (10%)**
- Syria	91 (29.6)	33	9	26	5
- Afghanistan	90 (29.3)	32	5	8	9
- Iraq	38 (12.4)	35	8	11	4
- Iran	34 (11.1)	35	9	14	4
- Albania	10 (3.3)	34	12	2	3
- Egypt	6 (2.0)	32	10	2	1
- Kosovo	4 (1.3)	31	12	–	2
- Serbia	3 (1.0)	34	3	–	–
- Moldovia	3 (1.0)	31	7	–	1
- Sudan	2 (0.6)	18	12	–	–
- Chechens	2 (0.6)	33	12	1	1
- Bosnia	2 (0.6)	36	4	–	–
- Eritrea	2 (0.6)	25	3	–	–
- Yemen	1 (0.3)	33	12	–	–
- Armenia	1 (0.3)	51	10	1	–
- no response	18 (5.9)	33	7	2	–

### Birth, pregnancy and abortion

The mean birth rate was 2.5 among participants at the time of the survey (range: 0–10). The majority of births were delivered naturally (70%), with 23% being performed by Cesarean section. Seventy-seven women reported to have experienced one or more miscarriages in their lives. Further data regarding parity and birthing method according to country of origin is shown in Table 2. Twenty-four women reported having had one or more abortions (range: 1–3 abortions per woman). No answer regarding abortion was given by 44 women. None of the women wished for an abortion at the time of the questionnaire.

**Table 2:** Parity and birthing methods (736 births)

Country of origin	Parity	Natural birth	C-section	Vacuum-assisted Vaginal delivery	Unspecified birthing method
		%	%	%	%
**Total**	**2.48**	**70**	**23**	**3**	**4**
- Syria	2.6	71	22	3	3
- Afghanistan	2.6	73	22	2	2
- Iraq	2.7	76	18	2	3
- Iran	1.9	58	25	5	10
- Albania	2.7	55	47	4	–
- Egypt	3.0	55	44	–	–
- Kosovo	2.3	77	22	–	–
- others	1.8	67	24	2	7

### Allocation to group 1

Twenty-four women (8%) stated that they were pregnant at the time of the survey whilst fifty-three women (17.3%) had a desire to become pregnant within the next 12 months. Twenty-three (7%) were already menopausal. Another 12 (4%) stated that they had never had a partner or had not recorded any history of sexual intercourse in the questionnaires.

### Allocation to group 2

**Fig. 1:**
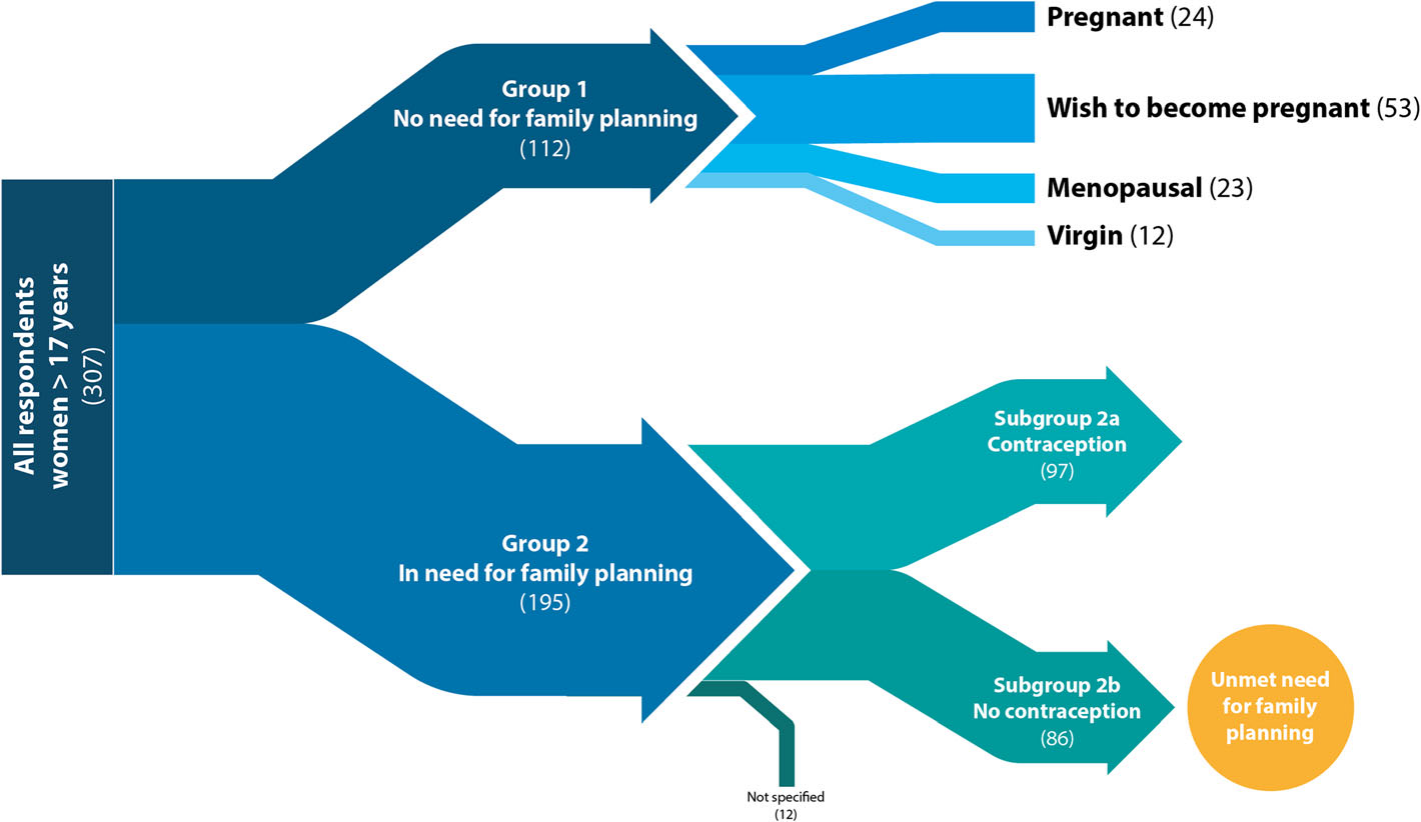
Group allocation (n)

Of the remaining 195 women with a theoretical interest in adequate contraception, 12 gave no information about their prevention method. We then further analyzed the remaining 183 women, of which 97 (53%) were using some form of contraception. Subsequently, we recorded an unmet need for family planning for 86 women (47% of women with a theoretical interest in adequate contraception). Those two groups were then further compared (Fig. 1).

### Subgroup comparison

Fertile women, who had an unmet need for family planning (subgroup 2b), were significantly older, with a median age of 36 years versus fertile women using contraception with a median age of 32 years (*p* = 0.001). Women using contraception (subgroup 2a) had been living in Germany significantly longer than women with an unmet need for family planning (median: 16 vs. 12 months; *p* = 0.013). Women who regarded themselves as sexually educated were significantly more likely to use a method of contraception than those who had no sexual education. (OR: 2.9, 95% CI: 1.27 to 6.72; *p* = 0.009) (Table 3 ).

**Table 3:** Comparison of unmet family planning needs versus contraception usage (*n* = 183)

Variables	Unmet need for family planning (Subgroup 2b) (***n*** = 86)	Contraceptive prevalence(Subgroup 2a) (***n*** = 97)	***p***-value
	n	n	
**Country of origin**			ns
- Albania	4	4	
- Syria	26	27	
- Afghanistan	23	33	
- Iran	8	12	
- Iraq	10	14	
- other	15	7	
**Age***Median*	*36*	*32*	0.001
**Months in Germany***Median*	*13*	*16*	0.013
**School**^a^			ns
- none	20	13	
- 1–5 years	7	9	
- 6–11 years	30	44	
- > 12 years	28	29	
**Felt sexually educated**	56	82	0.009
**Committed relationship**	50	82	0.001

^a^not specified by three women

*Abbreviaition*: *ns* not significant

### Subgroup 2a analysis

**Fig. 2:**
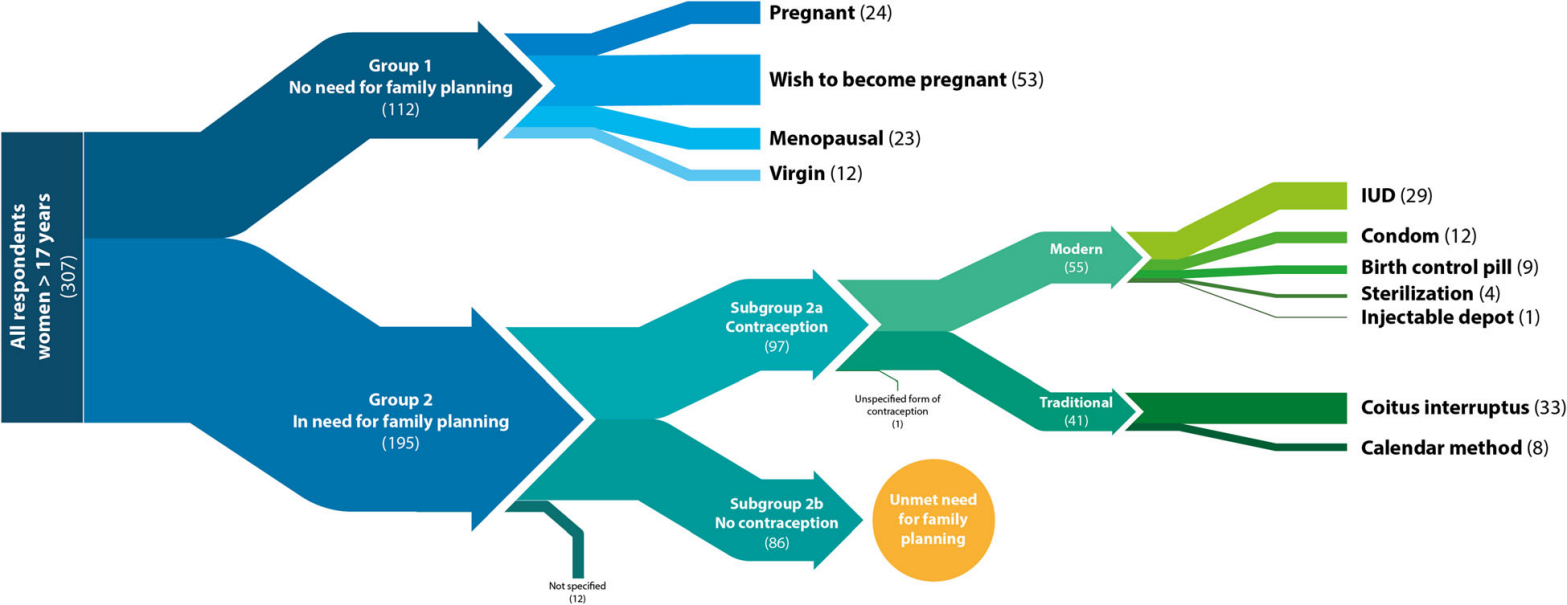
Methods of contraception (n)

The most commonly used methods of contraception were coitus interruptus (34%) and IUDs (30%). Seldomly used methods included condoms (12%), birth-control pills (9%), calendar method (8%), and sterilization (4%). In total 55% of women used “modern” methods such as birth-control pills, condoms and IUDs, while 42% of women used “traditional” methods like coitus interruptus or calendar method (see Fig. 2). The majority of women using these traditional methods came from Albania, Iran, and Iraq. However, whether the women opted for modern or traditional methods of contraception does not correlate with either sexual education, stay in Germany or present relationships as shown in Table 4.

**Table 4:** Comparison of contraceptive methods (*n* = 96)

Variables	Traditional (***n*** = 41)	Modern (***n*** = 55)	***p***-value
	n	n	
**Country of origin**			ns
- Albania	3	1	
- Syria	12	15	
- Afghanistan	11	21	
- Iran	7	5	
- Iraq	8	6	
- other	–	7	
**Age***Median*	*32*	*31*	ns
**Months in Germany***Median*	*14*	*16*	ns
**School**^a^			0.03
- none	4	9	
- 1–5 years	–	8	
- 6–11 years	20	24	
- > 12 years	17	12	
**Felt sexually educated**	34	47	ns
**Committed relationship**	37	44	ns

^a^not specified by two women

*Abbreviation*: *ns* not significant

Out of the women with an interest in family planning (group 2) 16 women already had at least one abortion, most of which came from Iran. At the time of being allocated into the subgroups, 11 of the 16 women (69%) were without any form of contraception and thus belonged to subgroup 2b. However, no statistical significance could be detected (*p* = 0.09).

## Discussion

### Unmet need among refugees

Most of the refugee women who participated in the study were young, without the wish to become pregnant any time soon and were sexually active. However, only half of them (53%) were using contraceptive methods, unlike women born and raised in Germany (rate of contraceptive usage among women who grew up and live in Germany: 68–91% [24]). Although refugees have the same rights and formal access to all means of contraception as German women [19], the unmet need for family planning in this group is high (47%).

Moreover, among the women using contraception, the withdrawal method (coitus interruptus) was most commonly used (34%) despite its inefficacy (Pearl-Index of 4–18) [25]. When refugee women opted for an effective method of contraception, they preferred the IUD (30%). This is unlike women born in Germany, who most often choose the birth control pill and condoms as their preferred contraceptive method [26]. Similarly to German women, our respondents were overall more likely to use some form of contraception when they were either in committed relationships or regarded themselves as sexually educated [27]. Interestingly, the choice between modern and traditional forms of contraception did not correlate with either relationship or sexual education. This indicates a gap in knowledge regarding the efficacy of different contraception methods.

The only information about the use of contraceptives that might be pertinent to refugees in Germany is a study performed by the World Bank. The authors evaluated contraceptive use among non-refugee women resident in the countries from which large portions of the refugees in Germany originate. The study supports our findings of an obvious access gap since the unmet need for family planning of refugees in Berlin shows to be higher compared to the results from the countries of origin (Afghanistan: 41% in Germany vs. 25% at home; Iran: 40% vs. 5.7%; Iraq 42% vs. 8%; Syria: 49% vs. 16.4%; Albania: 12.9% vs. 50%) [28].

### Reasons for the access gap

By law, refugees in Germany have the right to access any form of modern contraception free of charge [19]. However, the coverage for costs of contraception is state-specific and it needs to be arranged by health care professionals which can cause the process to often be cumbersome. In Berlin, female refugees can also obtain free contraceptives in the centers for sexual health and family planning, with no distinction made between the individual methods of contraception used. Despite this accessibility in Berlin, these services did not seem to reach our respondents. We propose this is due to a lack of information and health education services in Germany. This is based on studies that have shown that service quality is an important determinant of use of clinical contraceptive methods [16, 29]. Furthermore, language barriers can create an additional obstacle to those attempting to access services [11, 12, 18].

Another aspect worth considering is the self-reported diagnosis of depression among 84 women (27%). In comparison, in 2010 the prevalence of depression among German women was 14% [30]. These findings show the extent of the trauma which many of these women have suffered from their long migration history and could be another cause for some of their barriers to healthcare access and adequate contraception [7].

### Planned and unplanned pregnancies and their implications

In summary, due to the low rate of contraceptive use (subgroup 2b) and the high proportion of traditional methods with a higher pearl index within subgroup 2a, an increase in unplanned pregnancies can be expected if no further health policy measures are taken. The number of current pregnancies among our respondents resemble those of a study conducted at refugee camps in Lebanon and Iraq between 2014 and 2015 (8% of refugees in Germany vs. 7.5% in Lebanon and Iraq). Alarmingly, the same study stated that 57% of pregnancies were unplanned [31].

In our sample 7.8% of women reported having had one or more abortions to terminate an unwanted pregnancy. This is comparable to the termination rate in Germany (8.2%) [27]. Unintended pregnancies are known to be associated with a range of physical and psychological risks for mother and child as well as to increase barriers to access integration programs [7, 32]. This supports the evidence that all women regardless of their origin, upbringing and social status, need access to family planning services, including access to safe abortion services, in order to guarantee empowerment, successful integration and equity [33].

### Limitations

This study has several limitations to consider. Firstly, using a convenience sampling strategy may lead to potential selection bias. Nevertheless, due to the special living conditions of our respondents and the fact that this was a pilot study aiming to provide an overview of the current situation, we regarded this sampling method as adequate. Secondly, the very sensitive nature of the topic may have led to bias in completing the questionnaire. We tried to reduce this by making the discussion groups an event for women only. This is likely to have created a safe environment and a sense of trust and security amongst the participants. Furthermore, we made a considerable effort to ensure that our respondents understood that the researchers, gynecologists and interpreters were separate from the ministry of migration and had no influence on the decision on their refugee status. Nevertheless, participants may have felt that their answers could harm this process, affecting their willingness to complete the questionnaire or participate in the study. Although all eligible women were offered the opportunity to participate, women who already had their refugee status granted may have preferentially been included, since they may have shown a greater interest in participating.

The question of unplanned pregnancy remains a sensitive issue worldwide. The decision for a woman to use contraception, and what type of contraception, as well as the choice to terminate a current pregnancy, does not merely depend on her own opinion alone or her access to family planning. It also depends on her husband’s or partner’s involvement, her religion and culture [34] as well as her social status. These contributing factors were outside the scope of our study, as our aim was to give an initial overview of the current situation from which future studies can be based.

## Conclusion

Our study is the first to provide information about the current reproductive health status of female refugees in Berlin and gives an initial overview of their potential unmet need for family planning and contraception.

Family planning needs are high despite the fact that by law refugees are entitled to free access to any form of contraception. Nonetheless, an access gap is evident. The expected change in the number of planned and unplanned pregnancies will have a direct impact on the development of the German healthcare, education, and social system. Planned parenthood is a crucial factor for the successful integration of female refugees into their host countries. Therefore, a systematic family planning program focused on education and raising awareness for refugee women in Germany is essential.

In order to aim for stronger evidence on the development for targeted strategies, we have started a nationwide survey among female refugees in collaboration with the umbrella organization DaMigra. DaMigra conducts the same discussion groups in refugee community housing in four other cities in Germany. An extended version of our questionnaire is currently being used to cover family planning perceptions, and the role of the partner in decision making regarding contraception and health education. The results of our pilot study have formed the basis for such nationwide studies to evaluate the reasons and develop targeted strategies to bridge the access gap and empower refugee women within their own sexual and reproductive health.

## Supplementary information

**Additional file 1.** Questionnaire

## Data Availability

The datasets used and/or analyzed during the current study are available from the corresponding author on reasonable request.
