# Supplementary material for: Unmet family planning needs among female refugees and asylum seekers in Germany – is free access to family planning services enough? Results of a cross-sectional study
Source: Reprod Health. 2020 Jul 29;17:115. doi: 10.1186/s12978-020-00962-3 (PMC7389815; doi:10.1186/s12978-020-00962-3)
Supplement: Supplementary file 1 — Additional file 1. Questionnaire [file 12978_2020_962_MOESM1_ESM.docx]

Gynecological Questionnaire:

1. **General questions:**

| 1.1  What country are you from? |  |
| --- | --- |
| 1.2  Why did you flee your country? | Mortal Danger (1)  War (2)  Terrorism (3)  Torture (4)  Gender-specific persecution (5)  Health reasons (6)  c  Religious persecution (7)  Persecution due to being affiliated with a specific group (8)  Family already in Germany (9)  Impossible to secure a livelihood (10)  Deficient nutrition (11)  Other reasons ____________________ |
| 1.3  How old are you? | ___________ years old |
| 1.4  How long have you been in Germany? | ___________ months |
| 1.5  How many years of schooling have you had? | 0 ˂6 6 8 10 12 |
| 1.6  What vocational training do you have or what did you study? | Degree program started  Degree program completed  Field of study_______________  Vocational training starting  Vocational training completed  Type of vocational training _______________  No vocational training received |
| 1.7  Are you currently taking a German language course?  If not, then why not? | Yes  No, due to: Child care  Lack of organization at home  No self-confidence |
| 1.8  How tall are you and how much do you weigh? | _________ cm _________ kg |
| 1.9  Which of the following health conditions do you have? | High blood pressure (1)  c  Diabetes Mellitus (problems regulating your blood sugar levels) (2)  c  Depression (3)  c  Asthma (4)  c  Blood clots (5)  c  Other ________________________  c  None (0)  c |
| 1.10  Do you regularly take medications?  If so, which ones? | Yes, I take the following: _____________  ________________________  ________________________  ________________________  No |
| 1.11  Do you have allergies?  If so, to what? | Yes _______________________  _______________________  _______________________  No |
| 1.12  Do you regularly smoke?  If so, for how long and how many cigarettes per day? | Yes for approx. _________ years  around ________ cigarettes/day  No |
| 1.13  Do you regularly drink alcohol?  (at least once per day) | Yes  No |
| 1.14  Do you own a vaccination or health booklet detailing your immunization or medical history? | Yes  No |
| 1.15  Are you currently in a monogamous relationship? | Yes  No |

1. **General gynecological questions:**

| 2.1  Have you ever been to a gynecologist?  If so, when was your last visit? | Yes approx. _________ years ago  c  No, I have never been to a gynecologist. |
| --- | --- |
| 2.2  Are you currently going through menopause? If so, how old were you when you had your last menstrual period? | Yes approx. _________ years old  No, I still have my periods. |
| 2.3  If you answered “yes” to the previous question, have you had any medical complaints **following** your last menstrual period? | Yes Hot flashes (1)  Vaginal dryness (2)  Headache (3)  Anxiety (4)  Vaginal infection (5)  Other:_____________  No (0) |
| 2.4  Have you had any additional medical complaints **during** your menstrual period in the last 12 months? | Yes Lower abdominal pain (1)  Back pain (2)  Headache (3)  Nausea (4)  Poor blood circulation (5)  Very heavy bleeding –  more than 5 tampons  or sanitary pads per day  (Hypermenorrhea) (6)  Other  No |
| 2.5  Have you had abnormal symptoms during your menstrual period in the last 12 months? If so, which ones? | Yes Vaginal bleeding between periods  Absence of menstrual periods (Amenorrhea)  Prolonged menstrual bleeding >7 days (Menorrhagia)  Shortened menstrual bleeding <4 days  (Hypomenorrhea)  No |
| 2.6  Do you experience pain during sexual intercourse? | Yes Often  Sometimes  Seldom  No, never |

1. **Fertility**

| 3.1  Are you currently pregnant? | Yes  No I don’t know |  |  |
| --- | --- | --- | --- |
| 3.2  Would you like to become pregnant in the next 12 months? | Yes  No Maybe |  |  |
| 3.3  Do you currently use contraception? If so, what do you use? | Yes Condom (1)  Pill (2)  Shot/Injection (effective for 3 months) (3)  Coil/IUD (4)  Diaphragm (5)  Calendar method (6)  Coitus interruptus (7)  (withdrawal)  No (0) |  |  |
| 3.4  Are you aware of the various contraceptive methods? | Yes  No |  |  |
| 3.5  How often have you been pregnant and how have you delivered? | \| Pregnancy \| Natural birth \| Cesarean section \| Vacuum-assisted /  forceps \| \| --- \| --- \| --- \| --- \| \| 1. \|  \|  \|  \| \| 2. \|  \|  \|  \| \| 3. \|  \|  \|  \| \| 4. \|  \|  \|  \| \| 5. \|  \|  \|  \| \| 6. \|  \|  \|  \| |  |  |
| Please answer the following 3 questions, only if you have already been pregnant. | | | |
| 3.6  How many miscarriages have you had? | 0x 1x 2x 3x 4x 5x >5x |  |  |
| 3.7  How many voluntary abortions have you had? | 0x 1x 2x 3x 4x 5x >5x |  |  |
| 3.8  Were your pregnancies under the care of a doctor or midwife? | \| Pregnancy \| Doctor \| Midwife \| Without care \| \| --- \| --- \| --- \| --- \| \| 1. \|  \|  \|  \| \| 2. \|  \|  \|  \| \| 3. \|  \|  \|  \| \| 4. \|  \|  \|  \| \| 5. \|  \|  \|  \| \| 6. \|  \|  \|  \| |  |  |

1. **Gynecologic oncology**

| 4.1  Have you ever been diagnosed with cancer or suffered from cancer? If so, what type? | Yes with __________________  No, never |
| --- | --- |
| 4.2  Have you been screened for cervical cancer with a pap smear? If so, how many years ago was it? | Yes less than 1 year ago  more than 1 year ago  No, never |
| 4.3  Has your gynecologist screened you for breast cancer with a breast exam? | Yes less than 1 year ago  more than 1 year ago  No, never |
| 4.4  Do you regularly examine your breasts on your own? | Yes  No |
| 4.5  Have you been screened for breast cancer with mammography? (x-ray image of the breast) | Yes less than 2 years ago  more than 2 years ago  No, never |
| 4.6  Does cancer run in your family (mother, grandmother, sibling)? | Yes I don’t know  No |
| 4.7  Have you been vaccinated against HPV (cervical cancer)? | Yes I don’t know  No |

1. **Gynecologic infectious diseases**

| 5.1  Have you been diagnosed with HIV/AIDS? | Yes I don’t know  No |
| --- | --- |
| 5.2  Do you have knowledge of sexually transmitted infections? (HIV/AIDS, syphilis, gonorrhea, chlamydia, etc.) | Yes  No |

1. **Trauma**

| 6.1  Do you have nightmares? | Yes often  sometimes  seldom  No |
| --- | --- |
| 6.2  Have you personally experienced violence from others? If so, how often and from whom? | Yes often Husband/Partner  sometimes Family  seldom Acquaintance  Stranger  No, never |
| 6.3  Have you ever been sexually harassed/assaulted? If so, how often and from whom? | Yes often Husband/Partner  sometimes Family  seldom Acquaintance  Stranger  No, never |
